# Association Analysis of *METTL*23 Gene Polymorphisms with Reproductive Traits in Kele Pigs

**DOI:** 10.3390/genes15081061

**Published:** 2024-08-12

**Authors:** Jie Sun, Chunyuan Wang, Yan Wu, Jin Xiang, Yiyu Zhang

**Affiliations:** 1 Key Laboratory of Animal Genetics, Breeding and Reproduction in the Plateau Mountainous Region, Ministry of Education, College of Animal Science, Guizhou University, West Campus, Huaxi District, Guiyang 550025, China; 18096165277@163.com (J.S.); 18275126014@163.com (C.W.); 18785602686@163.com (Y.W.); m15186049094@163.com (J.X.); 2 Institute of Xiang Pigs, Guizhou University, West Campus, Huaxi District, Guiyang 550025, China

**Keywords:** *METTL*23, Kele pigs, single-nucleotide polymorphism, fertility traits

## Abstract

Methyltransferase-like 23 (*METTL*23) is a kind of RNA methyltransferase that catalyzes the methylation transfer to the N6-adenosine of RNA, serving as one of the key mediators in this process. However, the *METTL*23 gene has been poorly researched in pigs. In this study, we investigated the genetic effects of *METTL*23 single-nucleotide polymorphism(SNPs) on reproductive traits in Kele pigs. The DNA was extracted from 228 healthy multiparous Kele sows, and Sanger sequencing revealed three SNPs, g.4804958 G > T (intron 2), g.4805082 C > T (exon 2), and g.4806821 A > G (exon 3). The polymorphism information content (PIC) for each SNP was 0.264, 0.25, and 0.354, indicating moderate polymorphism (0.25 < PIC < 0.5) and providing genetic information. Linkage disequilibrium analysis showed no strong linkage disequilibrium between the three SNPs. The association analysis revealed that in the SNP g.4804958 G > T individuals with the GG genotype had a significantly higher number of piglets born alive, litter birth weight, number of weaned piglets, and weaning litter weight compared to those with the TT genotype (*p* < 0.05). Individuals with the GG genotype in the SNP g.4806821 A > G group had significantly higher litter birth weight and average birth weight than those with the AA genotype (*p* < 0.05). The H4H4 diplotype showed significant effects on the number of piglets born alive, litter birth weight, number of weaned piglets, weaning litter weight, and weaning weight (*p* < 0.05). Together, the *METTL*23 gene could be used as a candidate gene for the selection of reproductive traits in Kele pigs.

## 1. Introduction

Pigs were one of the earliest domesticated animals in the Near East around 9000 YBP [1,2]. China proved to be a major center of domestication in Asia [3]. The reproductive traits of sows determine the efficiency of pig production and economic returns, and one of the main reasons for weeding out sows is reproductive failure. It is an issue for economic and sustainability reasons [4], so the reproductive trait of sows has always been one of the most important concerns of breeders and the farming industry. As the reproductive trait is strongly influenced by the environment and traits with low heritability, we are limited to improving reproductive traits in pigs through conventional selection and crossbreeding systems [5]. The completion of the porcine genome sequence provided a valuable resource both in agricultural production and biomedical research [6]. One of the most important means to improve reproduction is the application of molecular breeding techniques in the selection of reproductive traits in pigs [7]. The Kele pig is one of the excellent local pig breeds in China, with its main origin in the Weining and Hezhang counties of Guizhou Province. It has the characteristics of cold resistance, roughage tolerance, high resistance to adversity, excellent meat quality with high increased intramuscular fat (IMF) [8], and well-developed hind legs, which is a high-quality raw material for making ham [9]. Due to the low reproductive performance of the Kele pigs, the development of the industry is restricted, and their reproductive performance needs to be improved. [10,11]

Methyltransferase-like 23 (*METTL*23) belongs to type I PRMTs and can catalyze the asymmetric dimethylation of histone H3R17 [12]. Protein arginine methyltransferases (PRMTs) are a class of enzymes that are responsible for adding methyl groups on arginine residues to target proteins [13,14]. This modification process, known as arginine methylation, is an important form of post-translational modification of proteins that affects a variety of biological processes, including gene transcription, cell signaling, protein interactions, etc. [15,16]. The family of PRMTs includes nine members, which have been classified into three categories: Type I, Type II, and Type III. Each PRMT member has different substrate specificities and methylation patterns, allowing them to perform their own unique functions in cells [17]. Studies in humans have found that the *METTL*23 gene mutation alters histone H3R17 methylation in normal-tension glaucoma [18]. Moreover, many of the studies on the *METTL*23 gene have been about human intelligence [19,20,21,22,23], and there were also studies showing that the *METTL*23 gene was associated with glaucoma in humans [24,25]. A study found that the *METTL*23 gene is implicated in congenital heart disease [26]. Another study has found that *METTL*23 is not the methyltransferase responsible for eEF1A methylation [27]. Yuki Hatanaka et al. [28] established that H3R17me2a and its catalyzing enzyme *METTL*23 are key regulators of paternal genome reprogramming. Wang et al. [29] identified that the *METTL*23 gene may play an important role in both the prognostic assessment and the prediction of recurrence risk for prostate cancer. In addition, studies on animals found that the *METTL*23 gene is involved in mental disorders in wild boars [30]. Furthermore, there was also a study about the *METTL*23 gene on medicinal plants, where Zhao et al. [31] discovered that the *METTL*23 gene has the potential to increase the production of dendrobium in the medicinal plant Dendrobium catenatum.

The porcine *METTL*23 gene is located on chromosome 12 (NC 010454.4) and encodes 190 amino acids with a total length of 6545 bp and five exons, and it is highly expressed in the ovary [32,33]. The *METTL*23 gene is a kind of type I PRMT, and studies have shown that the methylation modification by PRMTs plays an important role in mouse embryo implantation [34,35]. Research has demonstrated that protein arginine methyltransferases (PRMTs) are critically involved in murine embryogenesis, exerting regulatory control over gene expression, sustaining the pluripotency of stem cells, and facilitating the proper development of the nervous system [36,37,38]. However, the discovery of the *METTL*23 gene has had very limited reports in pigs. In this study, we hypothesized that the *METTL*23 gene affects reproductive performance on the foundation of previous research. For this purpose, we designed PCR primers for the coding region sequence (CDS) of the *METTL*23 gene and used Sanger direct sequencing to search for single-nucleotide polymorphisms (SNP) in the CDS of the *METTL*23 gene. In addition, the association analyses were carried out with the reproductive traits of Kele pigs in order to determine whether genetic variations in the *METTL*23 gene affect reproductive traits in pigs.

## 2. Materials and Methods

### 2.1. Experimental Materials

A total of 228 healthy sows were selected from the breeding farm of Kele pigs in Hezhang County, Bijie City, Guizhou Province, and reproductive indexes of two to four births were recorded, including piglets born alive, litter birth weight, average birth weight, number of weaned piglets, weaning litter weight, weaning weight, etc. A total of 0.5 g of ear tissue was collected from the sows with ear clamps, then we placed it into centrifuge tubes containing 75% ethanol for temporary storage and recorded the corresponding ear numbers. The ear tissues were taken back to the laboratory and stored in the refrigerator at −40 °C for future use.

### 2.2. DNA Extraction

The DNA was extracted using the tissue DNA kit (D3396-3) provided by Guizhou Xibao Trade Co., Ltd., (Guizhou, China) from the ear tissues of 228 Kele pigs. The concentration and purity of genomic DNA were detected by the nucleic acid concentration detector NanoDrop2000 (Thermo Fisher Scientific, Waltham, MA, USA) and checked at 1% agarose gel electrophoresis. Then, we stored the qualified samples at −20 °C.

### 2.3. Primer Design and Synthesis

The complete gene sequence of porcine *METTL*23 (NC_010454.4) was searched at the URL NCBI https://www.ncbi.nlm.nih.gov (accessed on 4 May 2024). The primer design excludes the first exon to circumvent non-specific amplification that may arise from its high GC content or sequence complexity, ensuring the precision and reproducibility of the experiment. Four pairs of primer sequences were designed around exon 2, 3, 4, and 5 regions of the *METTL*23 gene by Permer 5.0 software (listed in Table 1). The primers were synthesized by Bioengineering Co., Ltd. (Shanghai, China).

### 2.4. PCR Amplification

The PCR amplification system was a 20 μL reaction system, consisting of 10 μL 2 × Taq PCR Master Mix (GenStar, Beijing, China), 7 μL RNase-free H_2_O, 1 μL each of upstream and downstream primers, and 1 μL 10 μmol/L DNA template. The reaction procedure was set as follows, pre-denaturation at 95 °C for 8 min, denaturation at 95 °C for 30 s, annealing at 60 °C for 30 s, and extension at 72 °C for 30 s with 32 cycles. Final extension was at 72 °C for 8 min, then stored at 4 °C. After PCR amplification, products were detected by 1.5% agarose gel electrophoresis. The amplified products with good specificity were selected and sent to Sangon Biological Engineering Co., Ltd. (Shanghai, China).

### 2.5. Statistical Analysis

The genotype frequency, allele frequency, and polymorphic information content (PIC) of each site were calculated by using Excel 2020 to count all the mutation sites of the *METTL*23 gene. The Hardy–Weinberg equilibrium was analyzed by using the chi-square fitness test (χ^2^). The sequencing results of the *METTL*23 gene were screened and identified for SNP sites using the MegAlign program in the biological software DNAstar 17.6. The D’ values and R^2^ values for linkage disequilibrium of the SNP sites, as well as the haplotype frequencies, were calculated using the online SHEsis software http://analysis.bio-x.cn/ (accessed on 29 June 2024), and then the haplotype was used to synthesize the diplotype of each individual sow. IBM SPSS 23.0 (Armonk, NY, USA) was used to analyze the association analysis of *METTL*23 genotypes as follows:

y_ijk_ = μ+ G_i_ + P_j_ +G_i_ × P_j_ + e_ijk_ (1)

where y_ijk_ = the phenotyPIC observations, μ = the population mean, G_i_ = fixed effects of genotypes or diplotypes, P_j_ = fixed effects of different parity sows, G_i_×P_j_ = interactions between Gi and Pj, and e_ijk_ = random error. Data are presented as mean ± standard deviation (SD) and *p* < 0.05 as the criterion of significance of the differences.

## 3. Results

### 3.1. SNPs Identification

**Figure 1:**
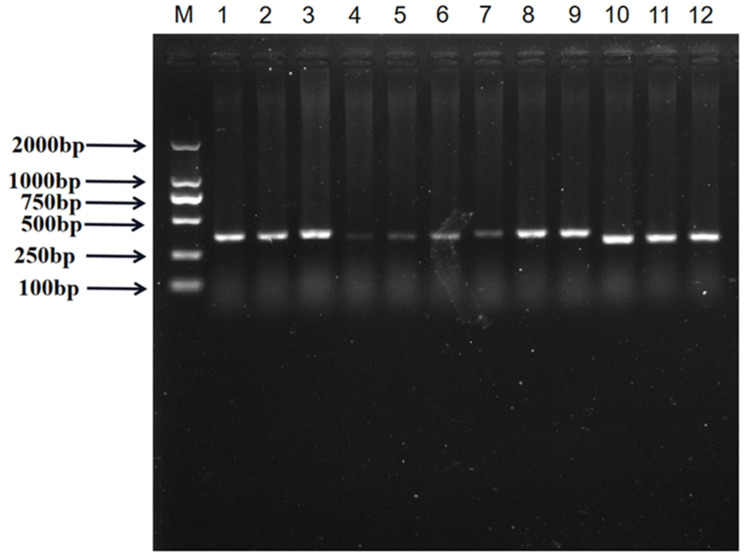
Agarose electrophoresis of PCR products of pig *METTL*23 gene, M, DL 2000 DNA marker; 1–3, P1 amplification product; 4–6, P2 amplification product; 7–9, P3 amplification product; 10–12, P4 amplification product.

**Figure 2:**
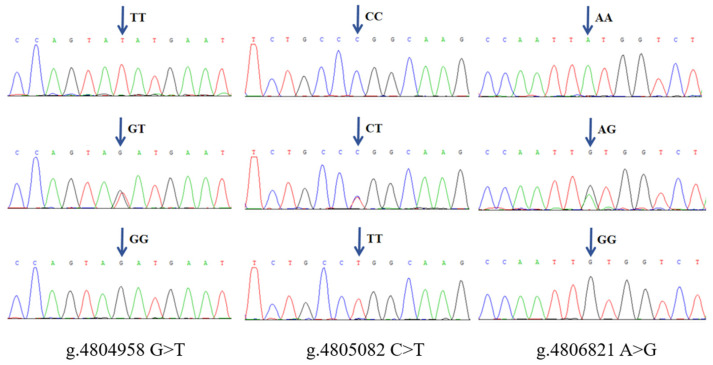
Sequencing alignment results of three SNPs in the *METTL*23 gene.

Agarose gel electrophoresis of PCR products showed that the amplified bands were clear, and the fragment sizes were consistent with the target fragments (Figure 1). Sequencing results of PCR products were further compared and analyzed. As shown in Figure 2, a total of three SNPs were found in the SNPs of g.4804958 G > T, g.4805082 C > T, and g.4806821 A > G, which existed in all three genotypes. Among them, the SNP g.4804958 G > T was located in intron 2, and the SNP g.4805082 C > T was located in exon 2. The coding amino acid codon had a mutation from CCC to CCT, and the coding amino acids were all proline (P), which was a synonymous mutation. Moreover, the SNP g.4806821 A > G was located in exon 3, the coding amino acid codon had a mutation from TTA to TTG, and the coding amino acids were all leucine (L), also a synonymous mutation.

### 3.2. Population Genetic Analysis of SNPs

The genetic characterization of the *METTL*23 gene’s SNPs in Kele pigs is shown in Table 2. The dominant genotypes in the SNPs g.4804958 G > T, g.4805082 C > T, and g.4806821 A > G were GG, CC, and AA, respectively. The corresponding dominant alleles for these genotypes are G, C, and A. The polymorphic information content (PIC) of the three SNPs was 0.264, 0.259, and 0.354, respectively, which were moderately polymorphic (0.25 < PIC < 0.5) and were therefore capable of providing a certain amount of genetic information. The results of the chi-square fitness test showed that the genotype distribution in the SNP g.4805082 C>T of the *METTL*23 gene was in agreement with the Hardy–Weinberg equilibrium (*p* > 0.05). The SNPs g.4804958 G > T and g. 4806821 A > G significantly deviate from the Hardy–Weinberg equilibrium (0.01 < *p* < 0.05).

### 3.3. Linkage Disequilibrium Analysis of SNPs

Linkage disequilibrium analysis of the three SNPs of the Kele pig *METTL*23 gene showed that (Table 3) among the three SNPs g.4804958 G > T, g.4805082 C > T, and g.4806821 A > G, none of them satisfy D’ > 0.800 and R^2^ > 0.330, which indicates that there was no strong linkage disequilibrium effect among the SNPs.

### 3.4. Haplotype and Diplotype Analysis of SNPs

Haplotype and diplotype analyses were carried out on the three SNPs of the *METTL*23 gene in Kele pigs (Table 4). The results showed that four haplotypes and ten diplotypes were detected. Haplotype H1 had the highest frequency (0.397), which was the dominant haplotype, and H4 had the lowest frequency (0.169), which was the inferior haplotype. Diplotype H1H2 had the highest frequency (0.202), which was the dominant diplotype, followed by H3H4 (0.105). In addition, H4H4 (0.026) had the lowest frequency, which was the inferior diplotype.

### 3.5. Association Analysis of SNPs in the METTL23 Gene with Reproductive Traits

By analyzing the parity effect, it was found that parity had no significant effect on the measured reproductive indexes. Further analysis showed that the SNP genotypes had significant differences in reproductive traits (shown in Table 5). The results showed that the number of piglets born alive, litter birth weight, the number of weaned piglets, and weaning litter weight of individuals with the GG genotype in the SNP g.4804958 G > T were significantly higher than those of individuals with the TT genotype (*p* < 0.05). The SNP g.4805082 C > T had no significant effect on the reproductive traits. The litter birth weight and the average birth weights with GG genotypes were significantly higher than those of AA genotypes *(p* < 0.05) in the SNPs g.4806821 A > G.

The association analysis between the three SNPs’ haplotypes and reproductive traits is shown in Table 6. The results indicate that the number of piglets born alive, litter birth weight, number of weaned piglets, and weaning litter weight from individuals of haplotype H4H4 (GGCCGG) were significantly higher than those of the other haplotypes (*p* < 0.05). Individuals of the haplotype H1H3 (GTCTAG) had a significantly higher average birth weight than individuals of the haplotypes H1H1 (TTCCAA), H1H2 (GTCCAA), and H4H4 (GGCCGG) (*p* < 0.05); and the weaning weight of individuals of haplotypes H4H4 (GGCCGG), H1H2 (GTCCAA), and H1H3 (GTCTAG) were significantly higher than that of H1H4 (GTCCAG), H2H2 (GGCCAA), and H3H3 (GGCTGG) diplotype individuals (*p* < 0.05). In conclusion, H4H4 was the optimal haplotype affecting reproductive traits in Kele pigs.

### 3.6. mRNA Secondary Structure Prediction of Different Haplotypes

**Figure 3:**
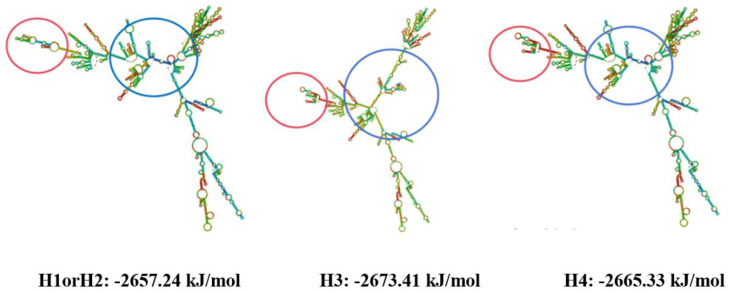
Comparison of secondary structure of mRNA of three haplotypes of *METTL*23 genes.

Two SNPs, g.4805082 C > T in exon 2 and g.4806821 A > G in exon 3, were selected to predict the secondary structure of the *METTL*23 gene in pigs using the online tool RNAfold http://rna.tbi.univie.ac.at/cgi-bin/RNAWebSuite/RNAfold.cgi (accessed on 30 June 2024). The mRNA secondary structures of each haplotype, predicted before and after gene mutation, correspond to the haplotype analysis presented above (Figure 3). Different parts of the secondary structures of the three mRNAs have been selected and highlighted with circles, and a total of two changes were observed. These observations indicated that the mutations at both loci caused changes in the mRNA secondary structure of the *METTL*23 gene. As in Figure 3, the SNP g.4805082 C > T caused the change in the blue circle, and the SNP g.4806821 A > G caused the change in the red circle. Haplotypes H1 and H2, corresponding to the genomic information included in NCBI, had a minimum free energy of −2657.24 kJ/mol. Haplotype H3 had a minimum free energy of −2673.41 kJ/mol. Haplotype H4 had a minimum free energy of −2665.33 kJ/mol. The combined changes in the secondary structure of the mRNA of the *METTL*23 gene, both the SNPs g.4805082 C > T and g.4806821 A > G, change the mRNA secondary structure, and both made the *METTL*23 gene mRNA structure more stable. 

## 4. Discussion

The reproductive traits of sows directly affect the productivity of pig farms and industrial economic returns. Compared with breeds that have high pig productivity and high lean meat percentages, such as Duroc, Landrace, and Yorkshire, Chinese local specialty breeds are at a disadvantage due to lower fertility, slower growth, and longer breeding cycles. Among the diverse pig breeds native to China, such as the Kele pig, several physiological traits are commonly observed. These include a relatively low litter size, precocious sexual maturity, a suboptimal reproductive rate, a notable tolerance to fibrous feed, adaptability to various stocking conditions, and a decelerated growth rate. It was found that there is a difference in germplasm between the Kele pig and the large white pig. Although the carcass quality of the Kele pig is not as good as that of the large white pig, its meat quality is excellent, with excellent breed characteristics such as strong water retention, juicy muscles, rich flavor [39], etc. These attributes render its meat highly desirable to consumers, underscoring the urgency of enhancing the Kele pig’s breeding traits as a critical issue in the field of porcine genetics.

Wu et al. [40] found that the *METTL*23 gene interacts with Ten-Eleven Translocation (TET) enzymes as an N6-methyladenosine (m6A)-modifying enzyme in DNA methylation and demethylation modification, proving its potential function in genome homeostasis regulation. Alarcón et al. [41] found that the *METTL*23 gene (also known as ZCCHC4) plays an important role as an m6A-modifying enzyme in methylation modification of RNA, revealing the interaction between it and HNRNPA2B1, which contributed to the comprehension of its function in RNA processing events in the nucleus. Zhao et al. [42] explored the effects of FTO-mediated m6A demethylation methylation on the regulation of mRNA splicing, and the *METTL*23 gene, as one of the m6A methyltransferases, was associated with RNA modification and splicing regulation. In light of these findings, this experiment held that methyltransferases might be involved in the methylation modification of RNA or DNA in cells to regulate gene (including reproductive performance-related genes) expression and cell biological processes, thereby affecting the reproductive traits of Kele pigs. In this experiment, the *METTL*23 gene was selected as a candidate gene for reproductive traits in Kele pigs, the SNPs of this gene were screened and identified by Sanger direct sequencing, and a total of three SNPs were found: g.4804958 G > T (intron 2), g.4805082 C > T (exon 2), and g.4806821 A > G (exon 3). The polymorphism information content (PIC) indicates the degree of population variation. High polymorphism is indicated by a PIC value greater than 0.5, which reflects a high degree of population variation. Moderate polymorphism is denoted by PIC values between 0.25 and 0.5, also indicating a high degree of population variation. Low polymorphism is indicated by a PIC value lower than 0.25, reflecting a low degree of population variation [43]. In this study, the polymorphic information content of the three SNPs varied, but all of them showed moderate polymorphism (0.25 < PIC < 0.5) with a large degree of population variation, indicating a certain amount of genetic information. The χ^2^ fitness test showed that the genotype distributions of the SNP g.4805082 C > T did not deviate from the Hardy–Weinberg equilibrium; this indicates that this locus has not been affected by mutation, selection, or genetic drift, or has been affected but is in a new equilibrium after long-term artificial selection and large-scale expansion. However, the SNPs g.4804958 G > T and g. 4806821 A > G significantly deviated from the Hardy–Weinberg equilibrium, and this suggested that the locus may be influenced by mutation, selection, and genetic drift. Linkage disequilibrium analysis is important for understanding the association between genes and phenotypes, revealing the genetic structure of human populations, and in the study of genetic diseases and complex traits, according to the basis for the division of linkage disequilibrium in gene seating as reported by Slatkin, Ardlie et al. and Andrade et al. [44,45,46], the SNPs g.4804958 G > T, g.4805082 C > T, and g.4806821 A > G did not have a strong linkage disequilibrium between each other (the conditions D’ > 0.800 and R2 > 0.330 were not satisfied).

In this study, the association analysis of the three SNPs of the *METTL*23 gene with reproductive traits showed that two SNPs, g.4804958 G > T and g.4806821 A > G, had a significant effect on the reproductive performance of Kele pigs. *METTL*23 is highly conserved among species and expressed in major organs such as porcine oocytes, testes, and early embryos. The *METTL*23 gene, a member of the protein arginine methyltransferase family (PRMTs), plays a crucial role in the regulation of early embryonic development and organ formation [47]. Research has demonstrated that the inhibition of protein arginine methyltransferases (PRMTs) in murine embryonic fibroblasts leads to hypo-methylation of glycine-arginine motifs, which in turn triggers spontaneous DNA damage and cell cycle dysregulation. This culminates in chromosomal aneuploidy or polyploidy, ultimately precipitating embryonic demise [48]. The aforementioned studies underscore the pivotal role of protein arginine methyltransferases (PRMTs) in the regulation of reproductive processes. The *METTL*23 gene is a type of PRMT. It may play an important role in the development of early embryos, tissues, and organs of pigs. In addition, there is a polymorphic site A62G in exon 4 of the *METTL*23 gene, which is significantly correlated with the number of litters of the Songliao black pig, and it can be used as a genetic marker for the number of litters of the Songliao black pig for molecular breeding. The number of piglets born alive, litter birth weight, the number of weaned piglets, and weaning litter weight in the GG genotype in the SNP g.4804958 G > T were significantly higher than those in the TT genotype (*p* < 0.05), while the litter birth weight and average birth weight in the GG genotype in the SNP g.4806821 A > G were significantly higher than those in the AA genotype (*p* < 0.05). Therefore, the GG genotype in the SNP g.4804958 G > T can be used for marker-assisted selection (MAS) to improve the traits of piglets born alive, litter birth weight, and the number of weaned piglets. Diplotype analysis has wide applications in genetics, biomedical, and population genetics studies. It provides an important tool for studying associations between genetic variation and phenotype, disease risk, drug reaction, etc. In addition, diplotype analysis is important for personalized medicine and genetic disease research. The correlation analysis between diplotypes and reproductive performance showed that the five indicators of reproductive data, respectively, piglets born alive, litter birth weight, number of weaned piglets, weaning litter weight, and weaning weight of diplotype H4H4 (GGCCGG) individuals were optimal across the group. In addition, the indicator of average birth weight of diplotype H4H4 (GGCCGG) individuals was above the average value of the group. In conclusion, diplotype H4H4 (GGCCGG) could be used as a marker-assisted selection (MAS) tool in breeding programs. Wan et al. [49] found that mRNA’s secondary structure had a direct impact on its expression, stability, and translation and then affects protein expression. Prediction of the mRNA secondary structure of the *METTL*23 gene of the Kele pig showed that mutations at the g.4805082 C > T and g.4806821 A > G loci would change the mRNA secondary structure of the *METTL*23 gene of the Kele pig, and both of them would make its minimum free energy smaller and more stable. The three SNPs are all synonymous mutations which lead to changes in the mRNA secondary structure, resulting in a more stable mRNA secondary structure. A more stable mRNA structure can increase the efficiency of protein translation and synthesis, thereby affecting its reproductive trait. However, the number of sows in this study, 228, is a limited sample size, which may lead to positive effects of SNPs on reproductive traits and thus limit the application of those SNPs. Therefore, we will further increase the sample to further identify these SNPs and discover new SNPs. At the same time, further studies of the biological function of the *METTL*23 gene through techniques such as gene silencing or overexpression will be carried out in order to lay the foundation for the utilization of the *METTL*23 gene.

## 5. Conclusions

In this study, a total of three SNPs were identified on the *METLL*23 gene of the Kele pig through Sanger sequencing, among which the SNP g.4804958 G > T had a significant effect on the number of piglets born alive, litter birth weight, number of weaned piglets, and weaning litter weight; and the SNP g.4806821 A > G had a significant effect on litter birth weight and average birth weight. The diplotype H4H4 (GGCCGG) had a significant effect on the number of piglets born alive, litter birth weight, number of weaned piglets, weaning litter weight, and weaning weight, indicating it can be used for marker-assisted selection (MAS). The *METTL*23 gene was able to be a candidate gene for reproductive traits in the Kele pig.

## Figures and Tables

**Table 1:** Primer information used for PCR.

Primers		Primer Sequences (5′→3′)	Amplified Fragment	Tm/°C
P1	F1	TGCTGGGCACTCAGTAATTGT	g.4804918~g.4805287 369bp/Exon2	60
	R1	AGACGCCACCAGTCCTGTTA		
P2	F2	AAGCCATTCACCAAGACCCG	g.4806377~g.4806725 348bp/Exon3	60
	R2	TCCATTGCATCGCTGGCTAA		
P3	F3	TGCCTGGAAATCTGTCAGCG	g.4806523~g.4806891 368bp/Exon4	60
	R3	ACACCAGTAAGGCTTCAGTTGAG		
P4	F4	ACTCAACTGAAGCCTTACTGGT	g.4806869~g.4807185 316bp/Exon5	60
	R4	AGCATTGACGCTGTCCTGAA		

**Table 2:** Population genetic information of three SNPs.

SNP Loci	Genotype Frequency			Allele Frequency		PIC	χ^2^
g.4804958 G > T	GG (93) 0.408	GT (89) 0.390	TT (46) 0.202	G 0.603	T 0.397	0.364	7.774 (0.02)
g.4805082 C > T	CC (150) 0.658	CT (70) 0.307	TT (8) 0.035	C 0.811	T 0.189	0.259	0.002 (0.99)
g.4806821 A > G	AA (103) 0.452	AG (87) 0.382	GG (38) 0.167	A 0.642	G 0.357	0.354	6.537 (0.03)

PIC is the polymorphic information content, χ^2^-HWE is the genotype Hardy–Weinberg equilibrium, χ_(2)_^2^ − 0.05 = 5.991, and χ_(2)_^2^ − 0.01 = 9.210.

**Table 3:** Linkage disequilibrium analysis of three SNPs.

SNP Loci	g.4804958 G > T	g.4805082 C > T	g.4806821 A > G
g.4804958 G > T	-	1.000	0.513
g.4805082 C > T	0.141	-	1.000
g.4806821 A > G	0.128	0.290	-

**Table 4:** Analysis of haplotype and diplotype of three SNPs.

SNPs		g.4804958 G > T	g.4805082 C > T	g.4806821 A > G	Frequency
Haplotypes	H1 (181)	T	C	A	0.397
	H2 (112)	G	C	A	0.246
	H3 (86)	G	T	G	0.189
	H4 (77)	G	C	G	0.169
Diplotypes	H1H1 (46)	TT	CC	AA	0.202
	H1H2 (38)	GT	CC	AA	0.167
	H1H3 (26)	GT	CT	AG	0.114
	H1H4 (25)	GT	CC	AG	0.110
	H2H2 (19)	GG	CC	AA	0.104
	H2H3 (20)	GG	CT	AG	0.088
	H2H4 (16)	GG	CC	AG	0.070
	H3H3 (8)	GG	TT	GG	0.035
	H3H4 (24)	GG	CT	GG	0.105
	H4H4 (6)	GG	CC	GG	0.026

**Table 5:** Association analysis of SNPs in *METTL*23 gene and reproductive traits in Kele pigs.

SNP Loci	Genotype	Piglets Born Alive	Litter Birth Weight/kg	Average Birth Weight/kg	Number of Weaned Piglets	Weaning Litter Weight/kg	Weaning Weight/kg
g.4804958 G > T	GG (93)	9.473 ± 2.224 ^a^	9.299 ± 3.154 ^a^	1.074 ± 0.221	7.484 ± 2.130 ^a^	39.459 ± 14.083 ^a^	5.257 ± 1.102
	GT (89)	8.653 ± 2.667 ^ab^	8.277 ± 2.614 ^ab^	1.070 ± 0.248	6.776 ± 2.477 ^ab^	38.206 ± 13.462 ^a^	5.794 ± 1.310
	TT (46)	7.568 ± 2.534^b^	6.403 ± 2.736 ^b^	1.021 ± 0.238	5.405 ± 2.327 ^b^	32.360 ± 15.524 ^b^	5.808 ± 1.301
g.4805082 C > T	CC (150)	8.887 ± 2.574	8.375 ± 3.171	1.044 ± 0.243	6.773 ± 2.425	37.941 ± 15.494	5.590 ± 1.301
	CT (70)	8.714 ± 2.583	8.489 ± 2.861	1.095 ± 0.221	6.986 ± 2.470	38.016 ± 11.852	5.622 ± 1.117
	TT (8)	8.250 ± 1.909	7.806 ± 0.895	1.175 ± 0.165	6.875 ± 1.808	32.363 ± 4.800	4.838 ± 0.605
g.4806821 A > G	AA (103)	8.659 ± 2.302	7.585 ± 2.563 ^b^	1.002 ± 0.248 ^b^	6.365 ± 2.230	35.362 ± 16.119	5.475 ± 1.396
	AG (87)	8.807 ± 2.417	8.612 ± 3.085 ^ab^	1.082 ± 0.220 ^ab^	6.952 ± 2.316	38.831 ± 12.402	5.689 ± 1.228
	GG (38)	9.1033 ± 3.047	9.223 ± 3.289 ^a^	1.125 ± 0.221 ^a^	7.367 ± 2.663	39.708 ± 13.415	5.567 ± 0.976

Different lowercase letters in the same column indicate significant (*p* < 0.05).

**Table 6:** Association of three SNPs in the *METTL*23 gene with reproductive traits in Kele pigs.

Diplotypes	Piglets Born Alive	Litter Birth Weight/kg	Average Birth Weight/kg	Number of Weaned Piglets	Weaning Litter Weight/kg	Weaning Weight/kg
H1H1 (46)	7.643 ± 2.792 ^c^	6.271 ± 2.050 ^d^	0.984 ± 0.235 ^b^	5.750 ± 2.102 ^c^	33.221 ± 13.936 ^c^	5.638 ± 0.683 ^ab^
H1H2 (38)	9.053 ± 1.930 ^b^	7.780 ± 2.673 ^cd^	0.982 ± 0.258 ^b^	6.605 ± 2.531 ^bc^	38.170 ± 17.678 ^bc^	5.776 ± 1.414 ^a^
H1H3 (26)	7.231 ± 2.386 ^c^	7.254 ± 2.491 ^cd^	1.233 ± 0.182 ^a^	5.461 ± 1.330 ^c^	34.508 ± 9.192 ^c^	6.482 ± 1.812 ^a^
H1H4 (25)	8.600 ± 2.769 ^b^	8.153 ± 2.424 ^c^	1.003 ± 0.157 ^ab^	7.120 ± 2.297 ^b^	36.588 ± 9.163 ^bc^	5.315 ± 0.913 ^b^
H2H2 (19)	9.368 ± 1.707 ^b^	9.128 ± 2.092 ^c^	1.071 ± 0.246 ^ab^	6.796 ± 1.584 ^bc^	32.900 ± 15.766 ^c^	4.632 ± 1.818 ^c^
H2H3 (20)	9.200 ± 1.056 ^b^	8.707 ± 2.566 ^c^	1.033 ± 0.270 ^ab^	7.450 ± 1.145 ^b^	40.882 ± 7.540 ^b^	5.497 ± 0.626 ^abc^
H2H4 (16)	10.750 ± 2.145 ^b^	11.325 ± 2.702 ^b^	1.114 ± 0.210 ^ab^	8.750 ± 1.949 ^ab^	48.431 ± 12.846 ^b^	5.507 ± 0.553 ^abc^
H3H3 (8)	8.250 ± 1.909 ^b^	7.806 ± 0.895 ^cd^	1.175 ± 0.165 ^ab^	6.875 ± 1.808 ^bc^	31.362 ± 4.800 ^c^	4.838 ± 0.605 ^c^
H3H4 (24)	8.583 ± 2.430 ^b^	8.265 ± 3.447 ^c^	1.069 ± 0.173 ^ab^	6.875 ± 2.863 ^bc^	35.231 ± 14.263 ^c^	5.342 ± 0.969 ^abc^
H4H4 (6)	12.500 ± 2.739 ^a^	12.533 ± 5.352 ^a^	0.997 ± 0.249 ^b^	9.667 ± 0.817 ^a^	57.933 ± 8.364 ^a^	5.983 ± 0.591 ^a^

Different lowercase letters in the same column indicate significant (*p* < 0.05).

## Data Availability

The authors affirm that all of the data necessary for confirming the conclusions of this article are present within the article, figures, and tables.
